# Impact of hot events at different developmental stages of a moth: the closer to adult stage, the less reproductive output

**DOI:** 10.1038/srep10436

**Published:** 2015-05-22

**Authors:** Wei Zhang, Xiang-Qian Chang, AryA. Hoffmann, Shu Zhang, Chun-Sen Ma

**Affiliations:** 1 Climate Change Biology Research Group, State Key Laboratory for Biology of Plant Diseases and Insect Pests, Institute of Plant Protection, Chinese Academy of Agricultural Sciences, Beijing, China; 2 Hubei Province Key Laboratory for Crop Diseases, Insect Pests and Weeds Control, Institute of Plant Protection & Soil Science, Hubei Academy of Agricultural Sciences, Wuhan, China; 3 Pest and Environmental Adaptation Research Group, School of BioSciences, Bio21 Institute, The University of Melbourne, Victoria, Australia

## Abstract

Hot days in summer (involving a few hours at particularly high temperatures) are expected to become more common under climate change. How such events at different life stages affect survival and reproduction remains unclear in most organisms. Here, we investigated how an exposure to 40 °C at different life stages in the global insect pest, *Plutella xylostella,* affects immediate survival, subsequent survival and reproductive output. First-instar larvae showed the lowest survival under heat stress, whereas 3^rd^-instar larvae were relatively heat resistant. Heat exposure at the 1^st^-instar or egg stage did not influence subsequent maturation success, while exposure at the 3^rd^-instar larval stage did have an effect. We found that heat stress at developmental stages closer to adult stage caused greater detrimental effects on reproduction than heat stress experienced at earlier life stages. The effects of hot events on insect populations can therefore depend critically on the timing of the event relative to an organism’s life-cycle.

Global climate change will not only lead to a substantial increase in average temperature but also in the frequency of hot events^1^. Maximum temperatures will often be high for short periods in summer in many regions around the world^2^,^3^,^4^. For example, summer daily maximum temperatures in *Brassica* fields at Wuhan (30.62N, 114.13E) in China often climb to 40 °C or more for several hours, and the number of such hot days has been increasing in the last 10 years (Fig. S1). Insects and other ectotherms in fields are increasingly likely to experience these short hot periods under global warming^5^,^6^. Species with relatively short generation times might experience hot days only at certain developmental stages. This raises the question of whether populations of organisms are susceptible to hot periods, which in turn will depend on the sensitivity of the life stage that is exposed.

Effects of hot events on demographics are likely to be stage specific because physiological responses to temperature change during development^7^,^8^. Despite recent progress in understanding the sensitivity of different developmental stages to heat stress measured by immediate survival in insects and other small invertebrates^9^, there is limited information on the longer-term consequences of hot events. Some studies have considered effects of heat stress in early stages on subsequent adult reproduction^10^,^11^,^12^,^13^,^14^. However, heat exposure on only one stage was usually considered, such as the egg stage^10^,^11^, 1^st^-instar larva^12^, or a more extended period of development^13^,^14^,^15^. The impacts of heat stress at different stages have only been compared in a few insects^16^,^17^.

Effects of heat stress are likely to differ depending on the nature of the species being considered. For instance, in aphids like *Metoplophium dirhodum* where the effects of heat stress have been considered^16^, there is the possibility that adults can repair and compensate the effects of heat stress at an earlier developmental stage through adult feeding. In contrast, Lepidoptera usually do not take in nitrogen sources at the adult stage, limiting the extent to which they might be able to repair damage due to heat stress at earlier developmental stages.

Here we investigate this issue in the diamondback moth, *Plutella xylostella*, the most destructive pest of cruciferous crops around the world. This species is well known for its ability to persist in stressed environments, such as under extreme high temperatures^18^,^19^,^20^, cold conditions^21^,^22^,widely fluctuating temperatures^10^ and in the presence of insecticides^23^,^24^. In our study site at Wuhan (30.62N, 114.13E), peak densities of *Plutella* usually occur from early May to early June and consist of multiple overlapping generations. During this time when hot events occur, all life stages are therefore present in the field.

We consider how 40 °C during different development stages of *Plutella* affects survival and reproduction. The following questions are considered. (1) Do heat effects on survival vary between development stages? (2) What is the impact of heat exposures at different stages on adult reproduction? (3) Does heat exposure in the most thermally sensitive stage (the most vulnerable to heat stress in terms of immediate survival) generate the largest reduction in adult egg production? To answer these questions, we investigated the effects of heat stress during five stages (egg, 1^st^-instar larva, 3^rd^-instar larva, pupa and both female and male adult) at 40 °C for 4–24 hrs exposures on immediate survival, subsequent survival to adulthood and fecundity.

## Materials and Methods

### Insect rearing

The population of *Plutella* larvae and pupae were originally collected on *Brassica* crops in fields at the experiment station of the Hubei Academy of Agricultural Sciences, China in May 2008. *Plutella* larvae were reared on artificial diet at 25 ± 1 °C, 60–80% RH and 15L: 9D, as described by Zhang *et al*. (2013)^18^. A total of ~6,000 eggs (≤6 hrs old) were collected for the experiment.

### Experimental protocol

We identified the effects of heat stress during five stages (egg, 1^st^-instar larva, 3^rd^-instar larva, pupa and both female and male adult) on immediate survival, subsequent survival (maturation success) and fecundity after exposure to 40 °C. Firstly, eggs were divided into six groups and each group was reared to different development stages at 25 ± 1 °C, 60–80% RH and 15L: 9D. For each stage, individuals were randomly assigned to six temperature treatments (40 °C for 4, 8, 12, 16, 20 or 24 hrs with 60–80% RH) or 25 °C in a climate chamber. Each temperature treatment involved 3 replications (eggs) or 4 replications (other stages). For each replication, we used 19–51 eggs (≤18 hrs old), 20–65 1^st^-instar larvae (newly hatched larvae,≤6 hrs old), 20–30 3^rd^-instar larvae (newly molt, ≤12 hrs old), 20–30 pupae (newly pupation,≤12 hrs old) or 10–20 females or males (newly emerged, ≤12 hrs old).

During heat stress, individuals were maintained in a Petri dish (9 cm diameter), and supplied with fresh artificial food (Southland Products Incorporated, USA) in the case of the larval treatment. Newly emerged females or males were stressed singly in a Petri dish (6 cm diameter). After heat stress, all individuals tested were removed to 25 °C for further rearing. Food was renewed every 3 days to assure that development and growth was not food limited. Once *Plutella* eclosed, a total of 16 surviving males and females from a given temperature treatment were paired, or all pairs were used if there were fewer survivors than 16 pairs. Adults were allowed to oviposit in a Petri dish (9 cm diameter), and held at 25 °C, 60–80% RH and 15L: 9D. Adults received a piece of fresh cabbage leaf (4 × 2 cm) for egg laying. Fresh cabbage leaves and Petri dishes were renewed every day. Temperature and humidity in the chamber were monitored (Taiwan Hengxin AZ Co., AZ-8829, Taizhong, China Taiwan); temperature variation for all treatments was within ±1 °C.

### Measurements

After heat stress, hatching status of eggs was checked (with a stereo microscope) twice daily at 08:00 and 20:00. For larva treatments, 1^st^-instar or 3^rd^-instar larval survival was checked after recovery at 25 °C for 24 hrs following the stress. Larvae were considered dead if their body did not move after touching them with a brush. The survivors of 1^st^-instar or 3^rd^-instar larvae after heat treatments continued to be observed daily at 08:00 am until all adults emerged. In pupal treatments, stressed pupae were checked twice daily until all adults emerged or pupae died (based on lack of adult emergence). For adult treatments, adult survival was checked 24 hrs after they were stressed. After pairing, we counted egg numbers (laid on the leaf and inner surface of the dish) daily across the first 7 days.

### Statistical analysis

All analyses were run with SAS V9.2. We compared immediate survival after heat stress between different development stages. For 1^st^-instar larvae, 3^rd^-instar larvae and adults, immediate survival was defined as the survival rate measured following a recovery at 25 °C for 24 hrs after heat stress. For eggs and pupae, immediate survival was measured as egg hatching success and pupal emergence rate when these stages were returned to 25 °C after heat stress. The relative impact of heat exposure of 40 °C at the different stages was assessed through the exposure time for 50% mortality (LT_50_ in hrs) to be observed. LT_50_ was estimated by fitting time–mortality data to logit models. LT_50_ values between the stage treatments were compared by examining their 95% confidence intervals. If the limits overlapped, lethal times were not considered to differ significantly^25^.

We explored the effect of heat exposures on subsequent survival (maturation success) after egg, 1^st^- or 3^rd^-instar larval heat stress. For egg treatments, maturation success was defined as the proportion of emerged adults from hatched eggs. For the 1^st^-instar or 3^rd^-instar larval treatment, maturation success was measured as the proportion of emerged adults from 1^st^- or 3^rd^-instars surviving after heat treatment. To compare treatments, we ran ANOVAs on survival data (with the proportion survival of each replicate group of eggs and larvae treated as data points). This was followed by post hoc Tukey B tests to determine which means differed significantly.

Adult egg production was determined as the total number of eggs laid across 7 days. Because egg production of the controls for the different developmental stages did not differ significantly, we compared heat treatments to a pooled set of controls. For each developmental stage, the relationship between exposure time and adult egg production was analyzed by fitting a linear regression model. We compared the slopes and intercepts of the regression lines by one-way analysis of covariance (ANCOVA)^26^. To further test the stage-specific heat effect on adult egg production, we compared the difference of adult egg production between different stage treatments in each exposure time, using one-way ANOVAs followed by Tukey B post hoc comparisons.

To compare the impacts of exposure time on the egg laying pattern, we calculated a midpoint of egg production (in days) for each individual, which is the period of time required for 50% of total egg production, For each stage, the relationships between midpoint of egg production and exposure time were analyzed through linear regression. A positive slope reflects postponed oviposition due to the heat stress, while a negative slope reflects an accelerated rate of oviposition.

## Results

### Immediate survival rate

**Figure 1:**
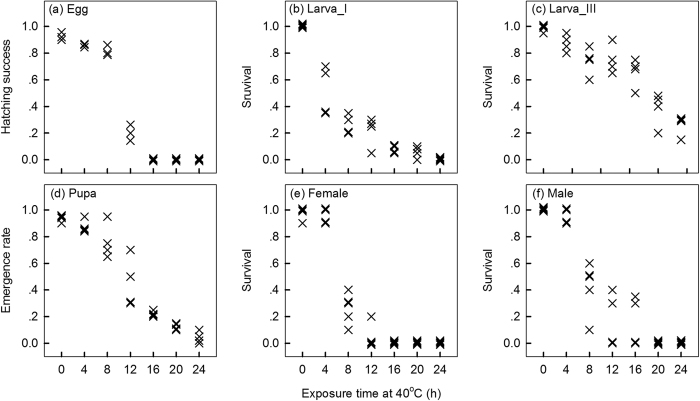
Immediate survival of each stage of *Plutella* after different exposures at 40 °C. Replications are shown separately. For (**b**) 1^st^-instar larva, (**c**) 3^rd^-instar larva, (**e**) female and (**f**) male, immediate survival is defined as the survival rate measured followed by a recovery at 25 °C for 24 hrs after heat stress. For (**a**) egg and (**d**) pupa, immediate survival is measured as the egg hatching success and pupal emergence rate respectively, when reared at 25 °C after heat stress.

**Figure 2:**
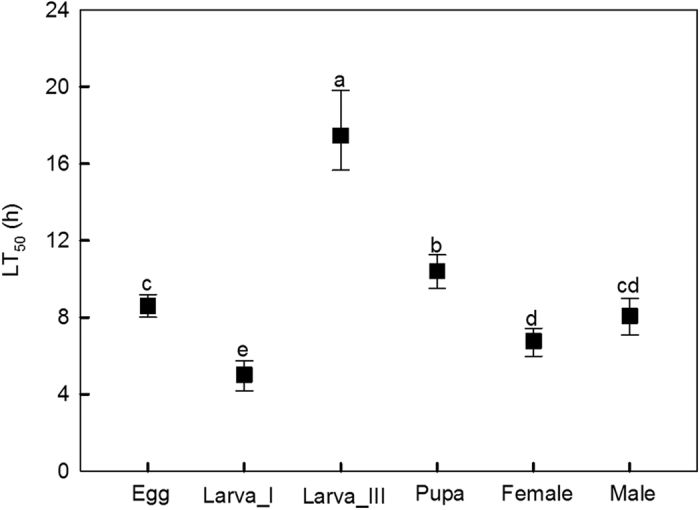
Lethal time (LT_50_ in hrs) and confidence interval (95% CI) of each stage of *Plutella* after heat exposure at 40 °C. Significant differences in LT_50_ were apparent between development stages of *Plutella* (indicated by non-overlapping 95% confidence intervals). Different letters indicate non overlapping confidence intervals.

Overall, survival rate declined with an increase of exposure time at 40 ^°^C at all stages (Fig. 1). Significant differences in LT_50_ were apparent between development stages of *Plutella* (indicated by non-overlapping 95% confidence intervals) (Fig. 2). The 3^rd^-instar larvae proved to be the most heat tolerant, with an LT_50_ of 17.5 hrs. Pupae had a relatively higher heat tolerance with an LT_50_ of 10.4 hrs followed by egg (8.6 hrs) and male adult (8.1 hrs) stages, and 1^st^-instar larvae were the least heat tolerant with an LT_50_ of 5.0 hrs. Female adults displayed a lower LT_50_ than male adults, although the 95% confidence intervals overlapped.

### Subsequent survival (maturation success)

**Figure 3:**
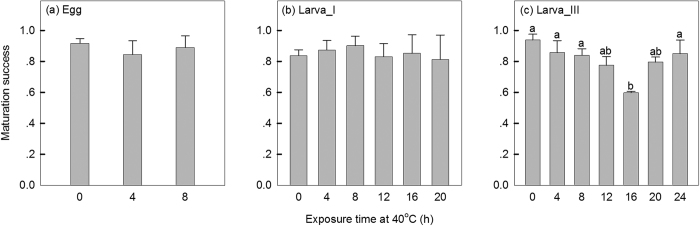
Maturation success (mean ± SE) after egg and larval exposures at 40 °C. Different letters above each bar indicate significant differences between exposure times at 40 °C (*P* < 0.05) based on post hoc tests. No eggs hatched when egg heat exposure exceeded 12 hrs. All 1^st^-instar larvae died after heat exposure for 24 hrs. Therefore maturation success could not be assessed for these treatments.

For egg treatments, heat exposure for 4 or 8 hrs did not result in a subsequent decrease in the number of adults that emerged from the hatched eggs compared to the control group, with a mean maturation success (±SE) of 0.88 ± 0.04 (Fig. 3a; F_2,6_ = 0.29, *P* = 0.756). Hatchlings from eggs exposed to 12 hrs heat stress could not survive to the adult stage. Heat exposures on 1^st^-instar larvae did not influence the number of adults that emerged from the surviving larvae (Fig. 3b; F_5,17_ = 0.13, *P* = 0.984). A complex pattern emerged for 3^rd^-instar larval treatments (Fig. 3c). As the exposure time increased from 0 to 16 hrs, maturation success decreased and was lowest (0.60) with the 16 hrs heat exposure (Fig. 3c; F_6,21_ = 4.38, *P* = 0.011). However, maturation success increased as exposure time increased from 16 to 24 hrs, with a high survival (0.85, N = 24) by those few that survived the 24 hrs treatment (Fig. 3c).

### Female egg production

**Figure 4:**
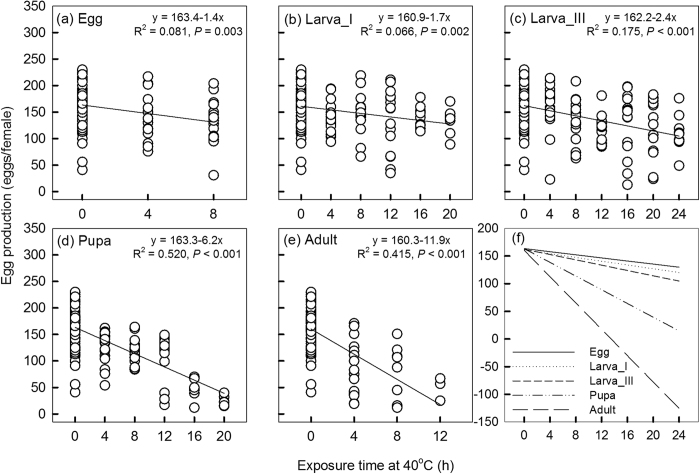
Relationship between egg production and exposure time at 40 °C of each development stage treatment. Linear regression lines and R^2^ values are included in (**a**)-(**e**). (**f**) Comparison of the slopes of regression lines from the different stage treatments.

**Figure 5:**
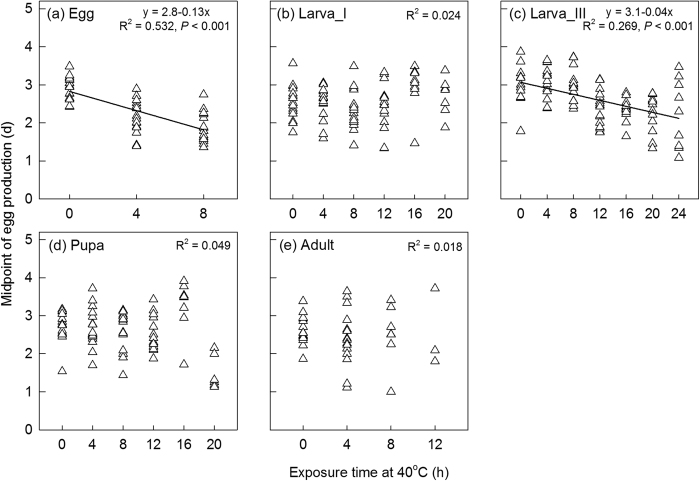
Relationship between midpoint of egg production and exposure time at 40 °C of each development stage treatment. The midpoint of egg production (in days) was determined as the period of time required for each individual to complete 50% of its oviposition. R^2^ values are included in (**a**)-(**e**) and linear regression lines are provided where patterns are significant.

Overall, egg production in the first 7 days declined with an increase in exposure time at 40 °C across all stages (Fig. 4). Although only conditions where sufficient numbers of individuals survived could be considered, there is a consistent pattern in that regression slopes become steeper as later developmental stages are heat stressed, resulting in significant differences between slopes (Fig. 4f; regression slopes: F_4,661_ = 21.41, *P* < 0.001; intercepts: F_4,661_ = 0.10, *P* = 0.981). The adult treatment showed the fastest decline in egg production when the duration of thermal stress increased, followed by the pupal treatment. The decline in egg and 1^st^-instar larva treatments appeared to be slower than for the 3^rd^-instar larva treatment, but we found no significant difference in slopes between these treatments (Fig. 4f, F_2,401_ = 1.61, *P* = 0.201). We examined different stage effects on adult egg production for each exposure time (Fig. S2). Stressed adults always produced the lowest egg numbers compared to pupal and larval treatments regardless of exposure time (Fig. S2, a-c, *P* < 0.019). Adults from the pupal treatment always laid lower numbers of eggs than those from the 1^st^- or the 3^rd^-instar larval treatment (Fig. S2, a, d, e, *P* < 0.002), although differences were not significant with exposures of 8 hrs (Fig. S2, b) or 12 hrs (Fig. S2, c). Heat treatment of 3^rd^-instar larvae tended to decrease egg production further than heat treatment of 1^st^-instar larvae, although these differences were small (Fig. S2, a-e). Regression analyses indicated no effect of exposure time on the midpoint of egg production of 1^st^-instar larval (Fig. 5b), pupal (Fig. 5d) or adult (Fig. 5e) treatments. However heat exposure time at the egg (Fig. 5a) and 3^rd^-instar larval treatments (Fig. 5c) did decrease the midpoint of egg production, reflecting faster egg laying by females from treatments with longer exposure times.

## Discussion

This study considered the impacts of hot events experienced at different development stages in *Plutella* moths; a period at 40 °C was used because this stress commonly occurs in agricultural production areas. To summarize the main findings, first instar larvae showed the lowest survival following exposure, whereas 3^rd^-instar larvae were relatively heat resistant. Heat exposure at early stages did not influence subsequent survival to adulthood (maturation success), while maturation success was affected by the 3^rd^-larval stage. Fecundity was affected most strongly by heat exposure at life stages closest to the adult stage rather than exposure at the earlier stage.

### Stage-specific survival under heat stress

Older instar larvae (or nymphs) often survive better than adults in many insects^27^,^28^,^29^,^30^. Older larvae may rapidly balance water loss through mass feeding^29^,^31^, while adult survival might be depressed if there is a trade-off between survival under stress and reproductive output^32^,^33^. Our finding that older larvae of *P. xylostella* showed higher heat tolerance than pupae or adults is also consistent with other *Plutella* studies which have shown this life stage to have low resistance under different protocols, including constant warm temperatures^34^ and heat shock in a ramping regime^19^, suggesting that the relative sensitivity of different ontogenetic stages may not depend on the method used to test for heat responses in *Plutella*.

Eggs, young larvae or pupae often have a relatively high level of heat tolerance. The pupae and eggs of *Drosophila buzzatii*^35^, *Otiorhynchus sulcatus*^36^ and *Wyeomyia smithii*^17^, as well as the early instar larvae of *Bombyx mori*^37^ and *M. dirhodum*^28^ are relatively tolerant of heat stress, perhaps because these stages have low mobility and are unable to evade a heat stress. In contrast, we found that first-instar larvae, eggs, and pupae of *Plutella* had relatively low heat resistance compared to other larval stages. This pattern has also been found for other *Plutella* studies^19^,^34^. Stress resistance may be affected by past selection pressures depending on the environment where different developmental stages are found^7^,^38^. *Plutella* eggs, first instar larvae and pupae usually occur on the underside of leaves^39^, where temperatures are cooler in hot days^40^,^41^, and this may help explain the relative sensitivity of these stages in contrast to the pattern in other insects.

### Heat stress effects on reproduction

The finding that heat stress on a life history stage closer to the adult stage is more detrimental for reproduction rather than heat applied at earlier stages may also apply to other taxa. Reproduction of the Homoptera insect, *M. dirhodum*, is reduced further by heat stress during late stages (4^th^-instar and adult stage) than early stages (2^nd^- and 3^rd^-instar)^16^. Egg production of the Diptera species, *W. smithii* appears to be depressed further by heat stress during the pupal stage than the egg or larval stages^17^. Lepidopteran studies involving *Plutella*^10^ and *Manduca sexta*^11^ also point to heat stress in the egg stage failing to influence adult reproduction. In addition to reproduction, morphological traits were impacted more by heat stress closer to adult stage than at earlier stages. In the Coleoptera species, *Harmonia axyridis,* adult body size and coloration were affected by heat stress at the 4^th^-instar larval or pupal stages but not at early development stages^42^. Moreover, in the butterfly, *Bicyclus anynana*, wing pattern was sensitive only to late larval stage temperatures^43^.

Stage-specific heat effects on reproduction might depend on whether enough time has elapsed for recovery to occur. Adult stresses often influence egg maturation and oviposition due to direct damage^44^,^45^. In addition, adult *Lepidoptera* may allocate less nitrogen and other resources to maturing eggs when they are stressed; stresses at the adult or late larval stage may reduce availability of these resources^46^,^47^, particularly as nutrition and water intake is required by insects to compensate for adverse conditions. Stresses on larval stages well before the adult stage may not have much impact on reproduction because holometabolous insects can repair and restructure morphology and physiological metabolism through metamorphosis^48^,^49^ to reduce the long-term effects on subsequent life-stages^10^,^11^.

Stressful conditions often induce adults to lay eggs as early as possible to avoid possible detrimental impacts later in life^50^. We found that heat stress applied to eggs or 3^rd^-instar larvae led to a faster rate of oviposition in *Plutella*. Body injuries have previously been reported to accelerate egg-laying^50^. Potential heat injury in eggs and 3^rd^-instar larvae might be carried over to the adult stage and lead to a more rapid rate of egg laying.

### Potential applications

Understanding how hot days affect population dynamics is likely to be important for pest management. To date, most studies on climate change have investigated effects of changes in mean temperature^51^ and fluctuating temperatures^52^ during an entire life cycle on organism performance. Ambient temperatures are rarely constant or rarely fluctuate in a fixed cycle. Instead in species with relatively short generation times, it is possible that any developmental stage might experience an extremely hot day.

Clearly the timing of hot events is likely to have important consequences for population size and local pest pressures involving *Plutella* due to effects on survival (immediate and through influencing maturation success) and reproduction. The number of surviving adults was reduced much more by a single hot event (e.g. 40 °C for 8 hrs) at the adult stage (>74%) than at other stages (<32%). In addition, reproduction was depressed furthest by a single hot event at the adult stage (55%), followed by pupal (28%), 3^rd^-instar (20%) and 1^st^-instar larval (12%) stages. If pests in a crop are mostly at late larval stage, heat stress may have less impact on population size because survival and reproduction are not affected much by such a stress. On the other hand, exposures at a later stage might depress population size and reduce pest pressures. Our results therefore highlight the importance of understanding how hot days affect survival and reproduction performance of the population with complex age/stage structure when predicting population dynamics under extreme events increasing in frequency under climate change.

## Additional Information

**How to cite this article**: Zhang, W. *et al.* Impact of hot events at different developmental stages of a moth: the closer to adult stage, the less reproductive output. *Sci. Rep.* **5**, 10436; doi: 10.1038/srep10436 (2015).

## Supplementary Material

Supplementary Information
