# Supplementary material for: Impact of hot events at different developmental stages of a moth: the closer to adult stage, the less reproductive output
Source: Sci Rep. 2015 May 22;5:10436. doi: 10.1038/srep10436 (PMC5377051; doi:10.1038/srep10436)
Supplement: Supplementary Information [file srep10436-s1.doc]

**Impact of hot events at different developmental stages of a moth: the closer to adult stage, the less reproductive output**

Wei Zhang^1^, Xiang-Qian Chang^2^, Ary A.Hoffmann^3^, Shu Zhang^2*^, Chun-Sen Ma^1*^

^1^Climate Change Biology Research Group, State Key Laboratory for Biology of Plant Diseases and Insect Pests, Institute of Plant Protection, Chinese Academy of Agricultural Sciences, Beijing, China

^2^Hubei Province Key Laboratory for Crop Diseases, Insect Pests and Weeds Control, Institute of Plant Protection & Soil Science, Hubei Academy of Agricultural Sciences, Wuhan, China

^3^Pest and Environmental Adaptation Research Group, School of BioSciences, Bio21 Institute, The University of Melbourne, Victoria, Australia

Figure S1 Anomalies in the number of hot days (DTmax ≥ 40^o^C) in June-August at Wuhan from 1980-2011. Daily maximum temperature records for 2013 were obtained from the China Meteorological Data Sharing Service System and the number of hot days appears to be increasing in recent times. Adapted from Zhang et al^1^.

Figure S2 Mean egg production (±SE) in the first 7 days after exposure to 40^o^C at different development stages. Each stage was exposed to a different time at 40^o^C (a-e). Different letters above each plot indicate significant differences (*P* < 0.05) between stage treatments based on Tukey B tests.

**Reference**

1. Zhang, W., Zhao, F., Hoffmann, A. A. & Ma, C. S. A single hot event that does not affect survival but decreases reproduction in the diamondback moth, *Plutella xylostella*. *PLoS ONE* **8**, e75923 (2013).
